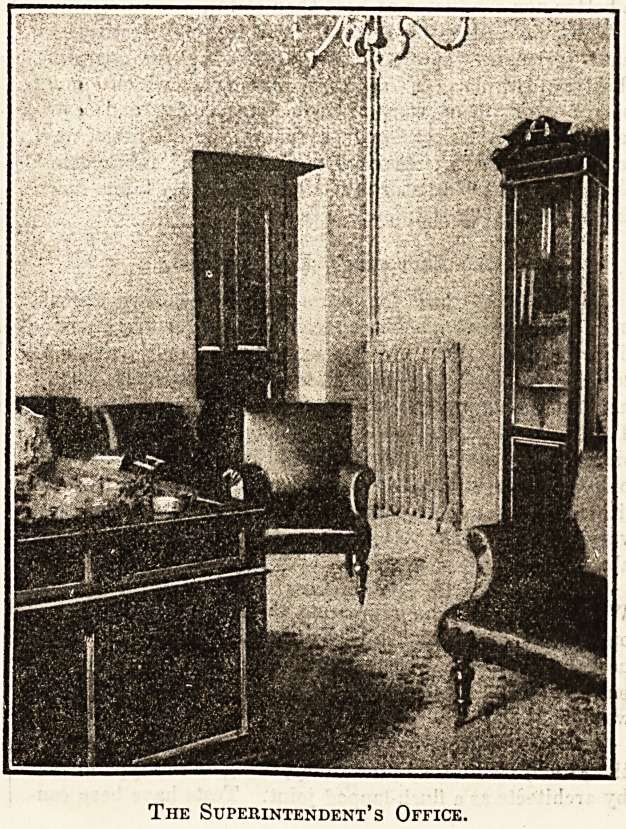# The Syngros Hospital, Athens

**Published:** 1912-02-17

**Authors:** 


### The Syngros Hospital, Athens.

By LASCARIS.

Athens is the capital of one of the smallest countries in Europe. It is therefore to the credit of its few wealthy inhabitants that it should be full of public institutions which are built and supported entirely by the charity of private individuals. So recently as August 12 (page 491) we noted the extensive hospital foundations projected in the will of the late Mr. Marino Corgialegno for the benefit of Athens.

The New Hospital.

Among the names of the great benefactors of their country none is more prominent than that of the late Andrea Syngros. Not content with introducing many improvements and spending lavishly on the Evangelismos Hospital, of which his wife was president, Mr. Syngros made a gift to the Poor Workwomen's Shop so substantial that it enabled the committee to start a splendid dining-room in the grounds of the building, where the workwomen can get a midday meal without leaving the spot or wasting time in preparing food. It would be tedious to enumerate all the philanthropical deeds of this citizen, whose grateful countrymen have called one of the principal roads of Athens after him.

One of the most substantial legacies for philanthropical purposes contained in the will of Mr. Syngros was for the erection and maintenance of a hospital for contagious diseases. His widow, the highly gifted Madame Syngros, took the greatest possible interest in every detail connected with the erection of this hospital. The planning of it was entrusted to the architect, Mr. Metaxas, who went into the plans and workings of all the most up-to-date hospitals in every country, and now this hospital is not merely the most up-to-date in Athens, but is allowed to compare favourably with all similar hospitals in and out of Europe by such authorities as Doctors Emery, Lacaspère, and Professor Bayet, of Brussels, the last of whom has visited every hospital of this nature all over the world, including Japan.

The front portion of the hospital consists of waiting and consulting rooms and a surgery for outdoor patients of both sexes, as well as a pharmacy, a micrological workshop, and a splendid amphitheatre, which, with its annexes and separate entrance, constitutes a separate department not communicating with the remainder of the building.

The corridors of this department are furnished with glass cupboards, containing casts, which are made on the premises under the direction of the professor who first introduced them into the country. The upper story of this department contains a room for the Röntgen rays, the d'Arsonval high-tension current, and the apparatus for treatment by electricity. There is also a room devoted to the Finsen rays for tuberculosis of the skin. This department also contains the paying patients' wards and the rooms of the doctors who live on the premises, as well as the beds for destitute male patients. At present there are only twenty-five of these beds, but we understand Madame Syngros intends adding to these, as the testator's object will never be carried out till treatment has been provided for men as well as for women.

The second department contains eight wards for women. These spacious wards are separated from each other by courts. Each ward contains twenty-five beds. There is a fine surgery and a room provided with basins for washing to every two wards. The hydropathic establishment and the water-closets, on approved new principles, are opposite these wards. The space between them is open for walks in summer and winter. The basement contains the prisons (*sic*), the disinfecting closet, the wash-house, and ironing rooms. The third department contains the church, a great white dining-room, and a kitchen with steam-cooking apparatus.

The whole of this hospital is kept most carefully, which is no small praise, considering the well-known multiplicity of dust in Athens.

The Superintendent's Office.

## Figures and Tables

**Figure f1:**
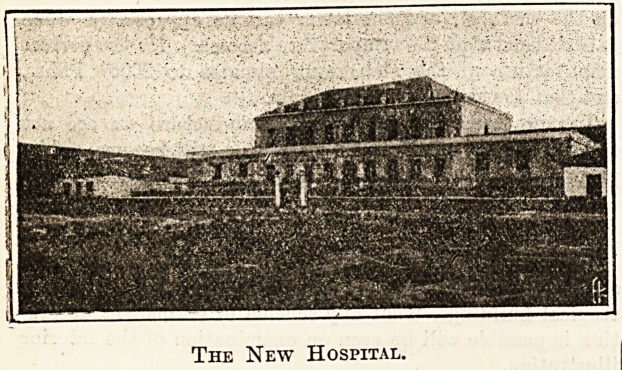


**Figure f2:**